# PCSK9 Loss‐of‐Function Disrupts Cellular Microfilament Network via LIN28A/HES5/JMY Axis in Neural Tube Defects

**DOI:** 10.1002/advs.202504291

**Published:** 2025-08-11

**Authors:** Xiaoshuai Li, Rui Wang, Wenting Luo, Hui Gu, Tianchu Huang, Qiushi Wang, Zhengwei Yuan

**Affiliations:** ^1^ NHC Key Laboratory of Congenital Malformation Shengjing Hospital of China Medical University Shenyang 110004 China; ^2^ Department of Stem Cells and Regenerative Medicine, Key Laboratory of Cell Biology, Ministry of Public Health, Key Laboratory of Medical Cell Biology Ministry of Education China Medical University Shenyang 110122 China; ^3^ Department of Blood Transfusion Shengjing Hospital of China Medical University Shenyang 110004 China

**Keywords:** cellular microfilament network, neural organoids, neural progenitor cells, neural tube defects

## Abstract

Neural tube defects (NTDs) are complex multigenic disorders and are the most prevalent and severe congenital malformations that affect the central nervous system. PCSK9 is identified as a molecular marker for the prenatal diagnosis of NTDs during its early stages in fetuses; however, its role in NTD neurulation and pathogenesis remains unclear. This study introduces PCSK9 knockout embryonic stem cells (ESCs) into neural organoid (NO) and neural progenitor cell (NPC) models and finds that PCSK9 loss leads to an incomplete neural tube structure in NOs and microfilament network disorder in NPCs. Transcriptome sequencing analysis shows that PCSK9 loss induces NTDs via the key molecule JMY. JMY overexpression in a zebrafish model increased the incidence and severity of PCSK9 loss‐associated NTDs. Mechanistically, PCSK9 acts as a molecular chaperone that promotes LIN28A degradation via the lysosomal pathway. LIN28A is an RNA‐binding protein that affects JMY expression by regulating the transcription factor HES5. Thus, PCSK9 loss disrupts the cellular microfilament network via the LIN28A/HES5/JMY axis, leading to NTDs. These findings provide important insights into the pathogenesis and therapeutics of NTDs.

## Graphical abstract

PCSK9 acts as a molecular chaperone promoting LIN28A lysosomal degradation. LIN28A elevates transcription factor HES5, increasing JMY expression. PCSK9 loss causes neural tube defects (NTDs) by disrupting the LIN28A/HES5/JMY axis, and high JMY disorganizes the neural progenitor cell microfilament network, leading to incomplete neural tube structure in organoids. This pathway reveals novel mechanisms and therapeutic targets for NTDs.

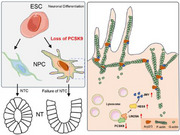

## 1 Introduction

Neural tube defects (NTDs) are the most prevalent congenital anomalies of the central nervous system.^[^ ^1^, ^2^, ^3^ ^]^ These disorders are complex and multigenic and occur as a result of the interplay between genetic predispositions and environmental influences.^[^ ^1^, ^2^, ^3^ ^]^ The annual incidence of NTDs ranges from 80 000 to 100 000 cases, and a majority of the affected children require lifelong medical management and care.^[^ ^1^, ^2^, ^3^ ^]^ The critical determinant of NTD incidence is failed neural tube (NT) formation,^[^ ^4^, ^5^, ^6^, ^7^, ^8^, ^9^ ^]^ which occurs during neural development through continuous proliferation, migration, differentiation, and morphological transformation of neuroepithelial cells.^[^ ^7^, ^8^, ^9^ ^]^ The neural plate bends to form a neural groove and eventually closes through intricate underlying mechanisms such as cytoskeletal alterations, cell‐to‐cell recognition and adhesion, changes in cell polarity, and programmed cell death.^[^ ^7^, ^8^, ^9^ ^]^ Failed NT closure (NTC) results in the development of NTDs.^[^ ^4^, ^5^, ^6^, ^7^, ^8^, ^9^ ^]^ Therefore, identifying the pathogenic genes that are responsible for the occurrence of NTDs because of NTC failure is essential for the early screening and targeted treatment of these defects.

The cellular microfilament network plays a pivotal role during NTC by maintaining the morphology of neuroepithelial cells and facilitating neural plate folding.^[^ ^10^, ^11^, ^12^ ^]^ During this process, the network exhibits a dynamic equilibrium.^[^ ^10^, ^11^, ^12^ ^]^ Disruption of this balance results in NTC abnormalities that may lead to NTDs.^[^ ^13^, ^14^, ^15^ ^]^ Additionally, the microfilament network is integral to the regulation of cellular processes such as migration, division, and differentiation as the proper execution of these cellular activities is vital for successful NTC.^[^ ^16^, ^17^, ^18^ ^]^ Knockout mice that lacked the cytoskeletal actin‐related genes SHROOM3 and P190RhoGAP exhibited NTDs.^[^ ^13^, ^15^ ^]^ Additionally, the overexpression of cytoskeletal actin results in NTDs.^[^ ^19^, ^20^ ^]^

Proprotein convertase subtilisin/kexin type 9 (PCSK9) was first identified in cerebellar neurons in 2003.^[^ ^21^ ^]^ Currently, PCSK9 is recognized for its two primary biological functions: regulation and maintenance of lipid homeostasis, neuronal differentiation, and apoptosis,^[^ ^22^, ^23^, ^24^ ^]^ of which its role in lipid homeostasis has been most predominantly addressed.^[^ ^22^, ^23^, ^24^, ^25^, ^26^ ^]^ PCSK9 primarily modulates plasma cholesterol homeostasis by specifically binding to members of the LDL receptor family and subsequently targeting them for degradation in the lysosomes, thereby inhibiting their normal circulation within the body.^[^ ^22^, ^23^, ^24^, ^25^, ^26^ ^]^ Human glial cells with silenced PCSK9 expression exhibited apoptotic characteristics.^[^ ^27^ ^]^ Furthermore, gene database analyses have shown a significant correlation between the functional loss of PCSK9 R46L and human NTDs,^[^ ^28^ ^]^ which suggests that PCSK9 is a crucial factor in nervous system development. We previously identified an association between PCSK9 and NTDs,^[^ ^29^ ^]^ although the extent of the direct correlation between PCSK9 loss and NTDs and the specific underlying pathogenic mechanisms remain unclear. Moreover, elucidating the PCSK9‐associated gene regulatory networks is imperative because NTDs are intricate polygenic disorders.

In this study, we developed neural organoids (NOs) from PCSK9 knockout (PCSK9^‐/‐^) embryonic stem cells (ESCs) to model NTDs and found that PCSK9 loss resulted in aberrant NT structures in the NOs. We subjected a transcriptome sequencing analysis and found that PCSK9 loss in the NPCs primarily caused the disorder of microfilament network assembly, which regulates the LIN28A/HES5/JMY axis during the early stage of neural development, leading to NT structural development. Furthermore, we used a zebrafish model and found that JMY overexpression exacerbated both the incidence and severity of PCSK9 loss‐associated NTDs. Thus, this study has elucidated a novel mechanism underlying the development of NTDs due to PCSK9 loss, which provides new perspectives for the early treatment of NTDs.

## 2 Results

### 2.1 PCSK9 Knockout Neural Organoid Model Exhibits Abnormal Structure and Reduced Dimension

**Figure 1:**
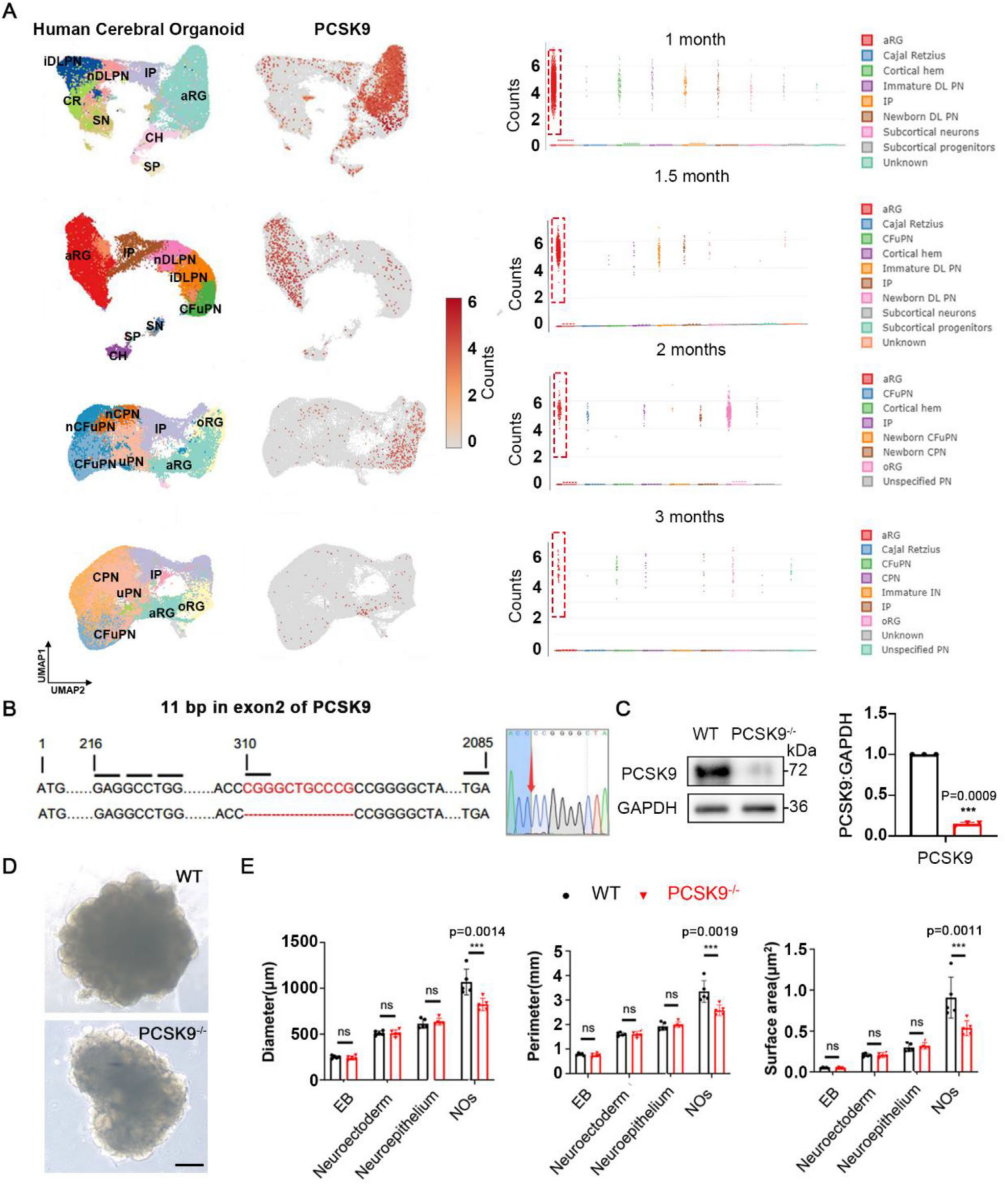
PCSK9 loss leads to changes in NOs size and NT structure. A) Cell‐type clusters, feature plot showing PCSK9 expression (left) and statistical results (right) in human cerebral organoids at different developmental time points. Dataset and cluster annotations were obtained from Uzquiano et al (2022), and the clustering and annotation from the original publication are kept unchanged. aRG: apical radial glia; oRG, outer radial glia; IP: intermediate progenitor; DL, deep‐layer; PN, projection neurons; iDLPN, immature DLPN; nDLPN, newborn DLPN; CFuPN, corticofugal projection neuron; CPN, callosal projection neuron; IN, interneurons; CR, cajal retzius; SN, subcortical neurons; CH, cortical hem; SP, subcortical progentors. B) CRISPR/Cas9‐mediated gene editing of the PCSK9 locus in ESCs resulted in an 11‐bp depletion in exon 2. C) Western blotting (WB) and quantification of PCSK9 expression in WT and PCSK9^‐/‐^ ESCs; n = 3 independent experiments. D) Representative images of WT and PCSK9^‐/‐^ NOs; scale bars, 1 mm. E) Quantification of diameter, perimeter, and surface area of NOs; n = 5 individual NOs. Values were mean ± SD. Statistical significance was determined using an unpaired two‐tailed Student's *t*‐test (C, E); ns: *P* > 0.05.

We analyzed a single‐cell database of human cerebral organoids at various developmental stages and found that PCSK9 expression was enriched in the first two months of brain development, with PCSK9 expression decreasing rapidly after the third month and becoming undetectable after the anaphase of brain development (**Figure** **1**A). Further analysis of cell subpopulations showed that PCSK9 is predominantly expressed in the apical radial glial (aRG) region with low expression in at least a fraction of intermediate progenitor cells and deep‐layer projection neurons and little expression in other neurons (Figure 1A). Therefore, we infer that PCSK9 plays a critical role in the development of the nervous system during the prophase of embryonic development.

To investigate the role of PCSK9 in nervous system development, we used the CRISPR‐Cas9 technology to generate PCSK9^‐/‐^ ESCs. We used information from the MIT website to predict and design guide RNAs (gRNAs) to target the second *PCSK9* exon (Figure S1A, Supporting Information). The knockout resulted in an 11‐bp deletion in exon 2, which caused a frameshift mutation that introduced a premature stop codon (Figure 1B). This mutation altered the protein structure beginning at the 104th amino acid (Figure S1B, Supporting Information) and affected the functional domain of the PCSK9 protein (Figure S1C, Supporting Information). PCSK9^‐/‐^ efficiency was confirmed through Sanger sequencing and Western blotting (WB) analysis (Figure 1B,C).

We investigated the effects of NTD‐associated PCSK9 loss using an NO system. The differentiation of wild‐type (WT) and PCSK9^‐/‐^ ESCs was induced through a four‐stage process (Figure S1D, Supporting Information); the corresponding light microscopy images are presented in Figure S1E (Supporting Information). To compare the formation of WT NOs and PCSK9^‐/‐^ NOs, an equal number of dissociated single ESCs (≈8000 starting cells) were aggregated to form embryoid bodies (EBs) during the initial stage. NOs were formed after an induction period of > 30 days (Figure 1D; Figure S1E, Supporting Information). Compared with that of WT NOs, PCSK9^‐/‐^ NOs exhibited reduced overall morphology with defective appearance (Figure 1D). Although both groups developed NT‐like structures, the NT structures in the PCSK9^‐/‐^ NOs showed incomplete development (Figure S1E, Supporting Information). Additionally, PCSK9^‐/‐^ NOs were smaller in size and were characterized by reduced circumferences and surface areas than those of the WT NOs (Figure 1E). These findings suggest that PCSK9 loss specifically affects NT structure and results in diminished NO size.

### 2.2 PCSK9 Loss Leads to Structural Disorder in NTs and Disorganized Cellular Microfilaments in NPCs

**Figure 2:**
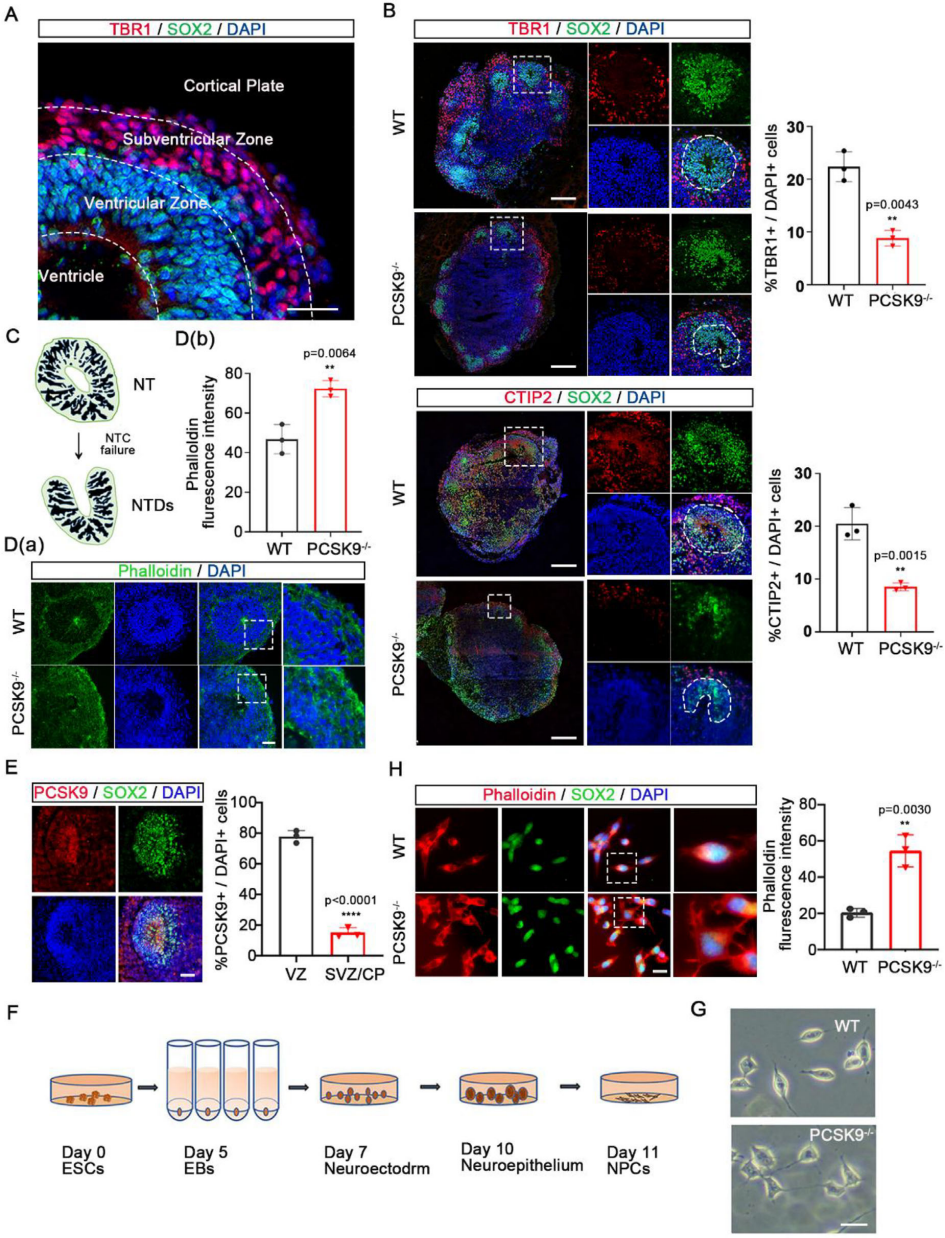
PCSK9 loss leads to disorganized cellular microfilaments in NPCs. A) NOs subjected to double immunofluorescence staining for TBR1 and SOX2 showed three distinct NT structures: the ventricle, ventricular zone (VZ), and subventricular zone/cortical plate SVZ/CP; scale bar, 100 µm. B) Immunofluorescence staining of TBR1+/CTIP2+ mature neurons in WT and PCSK9^‐/‐^ NOs. Representative images (left) and quantification (right) of TBR1+/CTIP2+ proportion in the mature neurons of the NT structure. The square shape indicates the NT structure, whereas the white circular or irregular shape represents the VZ region; n = 3 individual NOs; scale bar, 100 µm. C) Schematic diagram of NTDs caused by abnormal NTC. D) Immunofluorescent staining a) and fluorescence intensity quantification b) of cell microfilaments in the NT structures of WT and PCSK9^‐/‐^ NOs; n = 3 individual NOs; scale bar, 100 µm. E) Double immunofluorescence staining for PCSK9 and SOX2 showed the localization and expression of PCSK9 in the NT structure of WT NOs; scale bar, 20 µm. F) NPCs induction culture model map. G) Representative light microscopy images of WT and PCSK9^‐/‐^ NPCs; scale bar, 20 µm. H) Immunostaining and fluorescence intensity quantification of cell microfilaments in WT and PCSK9^‐/‐^ NPCs; n = 3 plates from WT and PCSK9^‐/‐^; scale bar, 100 µm. Values were mean ± SD. Statistical significance was determined using an unpaired two‐tailed Student's *t*‐test (B, D, E, H).

To facilitate a more detailed examination of the internal architecture, immunofluorescence staining was conducted on cross‐sections of NOs to elucidate their internal structure. SOX2 and TBR1 co‐labeling was used to differentiate NPCs from mature neuronal cells of the NOs. We found the development of NT‐like structures in three distinct regions of the NOs: the ventricle; ventricular zone (VZ), which is abundant in NPCs; and subventricular zone/cortical plate (SVZ/CP), which is rich in mature neurons (**Figure** **2**A). To verify whether PCSK9 loss results in NTDs, we identified various markers of mature neurons using immunofluorescence staining (TBR1: VI layer neurons, CTIP2: V layer neurons). WT NOs developed a complete NT structure with various mature neurons expressed and uniformly distributed around the NPCs. In contrast, the PCSK9 loss resulted in reduced expression of mature neurons in the NT structure and a relatively chaotic distribution of NPCs, which ultimately hindered the formation of a typical ring‐shaped NT structure (Figure 2B).

NTC failure during nervous system development leads to NTDs (Figure 2C). The abnormal expression and distribution of cytoskeletal proteins contribute to NTC failure.^[^ ^10^, ^11^, ^12^ ^]^ We used phalloidin dye to perform immunofluorescence staining of the NO cellular microfilaments (Figure 2D(a)). Compared with that of the WT group, the cells comprising the NT structure of the PCSK9^‐/‐^ NOs exhibited a disordered distribution and increased expression of cellular microfilaments, which is consistent with NTC failure (Figure 2D(b)). Additionally, we validated the association between PCSK9 dysfunction and NTDs in humans by generating PCSK9^R46L^ mutation^[^ ^28^ ^]^ and NTD‐associated VANGL2^‐/‐[^ ^30^, ^31^ ^]^ human induced pluripotent stem cells (iPSCs) and differentiated them into NOs (Figures S2 and S3, Supporting Information). Immunofluorescence staining showed that both PCSK9^R46L^ and VANGL2^‐/‐^ NOs exhibited reduced expression of mature neurons in NT‐like structures compared with those of the WT group and failed to form a typical ring‐shaped NT structure (Figures S2C and S3C, Supporting Information), similar to the PCSK9^‐/‐^ NT structures.

Analysis of a single‐cell database of human cerebral organoids showed that PCSK9 was predominantly localized in the aRG region during neural development (Figure 1A). As aRG cells are an NPC subset, this finding suggests specific PCSK9 enrichment in this NPC subpopulation. To validate this hypothesis, we performed immunofluorescence staining of the WT NOs. PCSK9 was predominantly enriched in the SOX2+ NPC subpopulation in the NT group (Figure 2E). Its absence may cause the aberrant differentiation of NPCs, which ultimately results in an abnormal NT structure.

To investigate the effect of PCSK9 loss on NPC‐related cellular behavior, both WT and PCSK9^‐/‐^ ESCs were subjected to a three‐stage induced differentiation process (Figure 2F), followed by dissociation into single cells for NPC culture. The WT NPCs exhibited a long and spindle‐shaped morphology, whereas the PCSK9^‐/‐^ NPCs exhibited abnormal, disordered, and multibranched structure (Figure 2G), which is typically attributed to the aberrant expression of cytoskeletal proteins. Hence, we stained the cellular microfilaments in NPCs. As shown in Figure 2H, the PCSK9^‐/‐^ NPCs exhibited a disordered morphology with the majority of cells exhibiting a multibranched appearance and increased fluorescence intensity. Overall, these findings suggest that PCSK9 loss results in the disorganization of cellular microfilaments in NPCs.

### 2.3 PCSK9 Affects NT Structure by Regulating Microfilament Network Formation in NPCs through JMY

**Figure 3:**
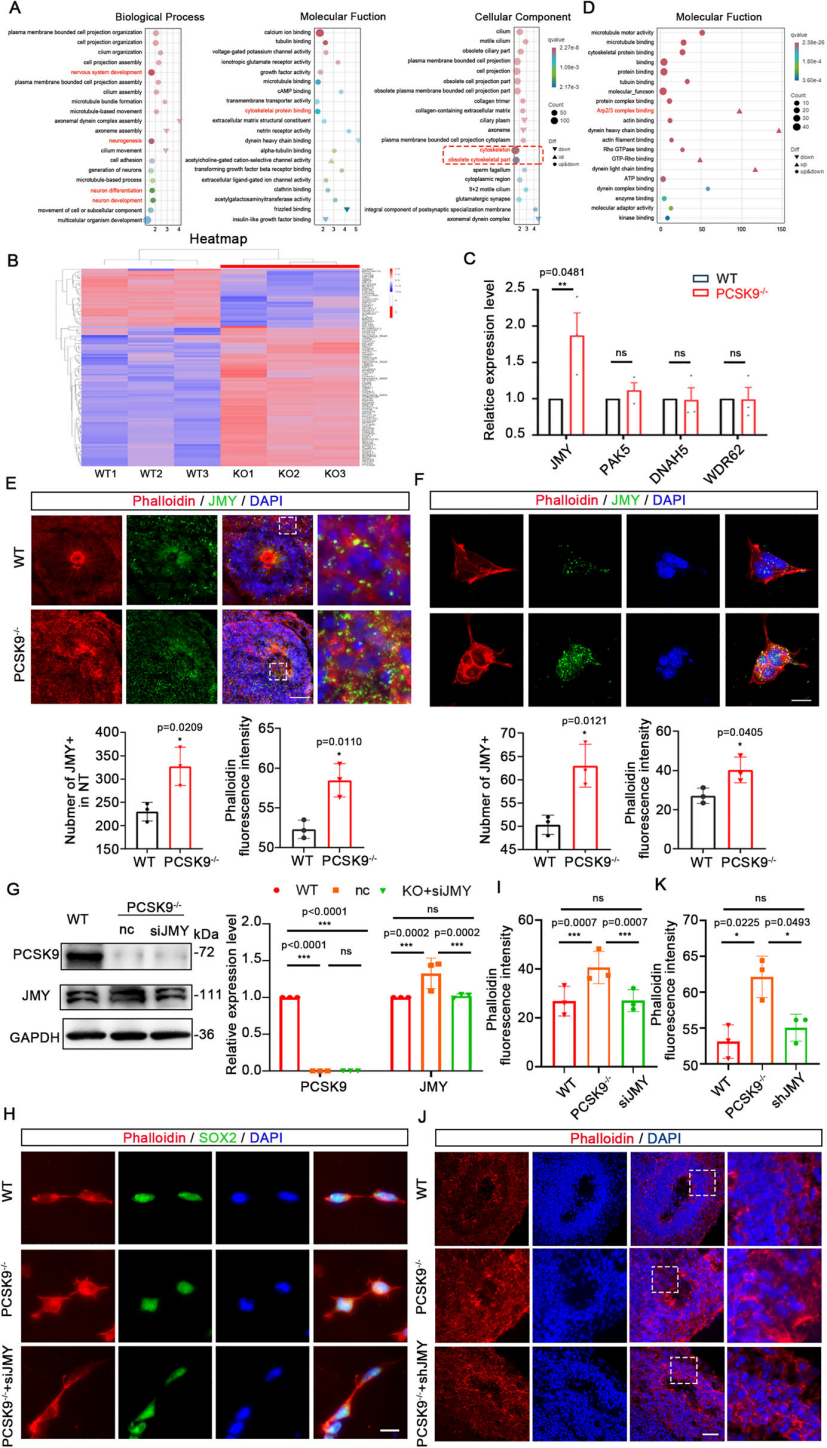
PCSK9 affects NT structure by regulating microfilament network formation in NPCs through JMY. A) Biological process‐, molecular function‐, and cellular component‐related items in the GO analysis. The red box shows the cytoskeletal and obsolete cytoskeletal part‐related items. Different colors represent different q values, ranging from red to blue, with q values increasing from small to large; the smaller the q value, the better the correlation. Graph size represents the number; a larger graph indicates more altered genes. In the entry, ▼ indicates downregulation of the altered gene; ▲ indicates upregulation of the altered gene; and ● indicates both upregulation and downregulation of the altered genes. B) Heat map of all DEGs in cytoskeletal proteins. The three rows on the left are the WT group, and the three rows on the right are the PCSK9^‐/‐^ group. Red indicates high expression, and blue indicates low expression. C) Statistical results of qPCR validation of the altered genes. D) Molecular function‐related items of cytoskeletal proteins. The red box shows the Arp2/3 complex binding entry where JMY is located. E,F) Immunofluorescence staining (up) and statistical results (down) of the expression and colocalization of JMY and cell microfilaments on the NT structure of NOs (E) and NPCs (F); n = 3 individual experiments; scale bar, 100 µm (E), 10 µm (F). G) WB and quantification of PCSK9 and JMY protein levels in WT and PCSK9^‐/‐^ NPCs after siJMY rescue experiments; n = 3 individual experiments. H,I) Immunofluorescence staining and fluorescence intensity quantification of cell microfilaments in WT and PCSK9^‐/‐^ NPCs with and without siJMY rescue; n = 3 plates from WT, PCSK9^‐/‐,^ and PCSK9^‐/‐^ +siJMY; scale bar, 20 µm. J,K) Immunofluorescence staining and fluorescence intensity quantification of cell microfilaments in WT and PCSK9^‐/‐^ NT structure with and without shJMY rescue; n = 3 individual NOs; scale bar, 100 µm. Values were mean ± SD. Statistical significance was determined using an unpaired two‐tailed Student's *t*‐test (C, E, F). Statistical significance was determined using one‐way ANOVA with the Dunnet post hoc test (G, I, K); ns: *P* > 0.05.

To investigate the causes and potential mechanisms underlying NT structural abnormalities resulting from PCSK9 loss, we utilized transcriptome sequencing methods to elucidate the underlying causes. There were of 3764 differentially expressed genes (DEGs) were identified via transcriptomics after quality control. Of these, 2392 are upregulated and 1372 are downregulated. Gene ontology (GO) analyses of the DEGs between WT and PCSK9^‐/‐^ showed specific enrichment of selective pathways. Furthermore, GO analysis of these biological processes showed that the DEGs were predominantly associated with nervous system development, neurogenesis, neuron differentiation, and neuron development. The molecular functions of these DEGs were primarily related to cytoskeletal protein functions. Similarly, the cellular components of the DEGs were primarily associated with cytoskeletal proteins (**Figure** **3**A). These results indicate that the DEGs are predominantly associated with neural development and cytoskeletal proteins. This correlates with our findings that aberrant expression and distribution of cytoskeletal proteins result in NTDs.

We identified the key genes associated with the abnormal cytoskeletal proteins resulting from PCSK9 loss by analyzing the DEGs associated with the cytoskeleton and obsolete cytoskeletal parts of the cellular components. In total, 140 DEGs were implicated in these cytoskeletal protein alterations (Figure 3B). Subsequently, we validated four genes with significant changes using quantitative PCR (qPCR) and found that the alteration in JMY expression was statistically significant (Figure 3C). Analysis of the 140 DEGs showed that JMY participated in the assembly of the Arp2/3 complex, which is associated with cellular microfilaments, and this involvement was notably upregulated (Figure 3D). Therefore, we hypothesized that PCSK9 loss results in an aberrant increase in JMY expression, which influences the organization of the cellular microfilament network by modulating the assembly of the Arp2/3 complex, thereby affecting cell morphology.

We investigated the effect of PCSK9 knockdown on JMY expression and localization in NOs by performing immunofluorescence staining. The results showed that WT NOs exhibited intact NT structures, with JMY predominantly localized to the microfilaments and exhibiting normal expression levels (Figure 3E). In contrast, PCSK9^‐/‐^ NOs exhibited NTs that were not completely enclosed, resulting in a widespread distribution of JMY within the nucleus and cell microfilaments. Furthermore, JMY expression significantly increased and was clustered at the gap of the NT structures (Figure 3E). Statistical analysis indicated that the JMY level in the PCSK9^‐/‐^ group was elevated and accompanied by the increased expression of cell microfilaments (Figure 3E). We investigated the NPCs and observed that the PCSK9^‐/‐^ NPCs showed irregular morphology with high expression of JMY, which was extensively distributed in the nucleus and microfilaments (Figure 3F). Subsequent rescue experiments confirmed that JMY positively regulated the expression of cell microfilaments. Furthermore, suppression of JMY expression during the differentiation of PCSK9^‐/‐^ NPCs showed the amelioration of the morphological abnormalities in NPCs, which were characterized by increased branching (Figure 3G–I). Subsequently, we validated the NOs and found that JMY knockdown restored most of the structures in PCSK9^‐/‐^ NOs to ring structures and restored the expression of cell microfilaments (Figure 3J,K; Figure S4, Supporting Information). Immunofluorescence quantification showed a significant increase in mature neuronal markers in JMY‐knockdown mutants compared with those in the PCSK9^‐/‐^ group (Figure S4, Supporting Information). Phenotypic rescue suggests that JMY dysregulation is a critical downstream effector of PCSK9 loss that potentially acts by disrupting cytoskeletal remodeling.

### 2.4 HES5 is a Transcription Factor that Regulates JMY Expression

**Figure 4:**
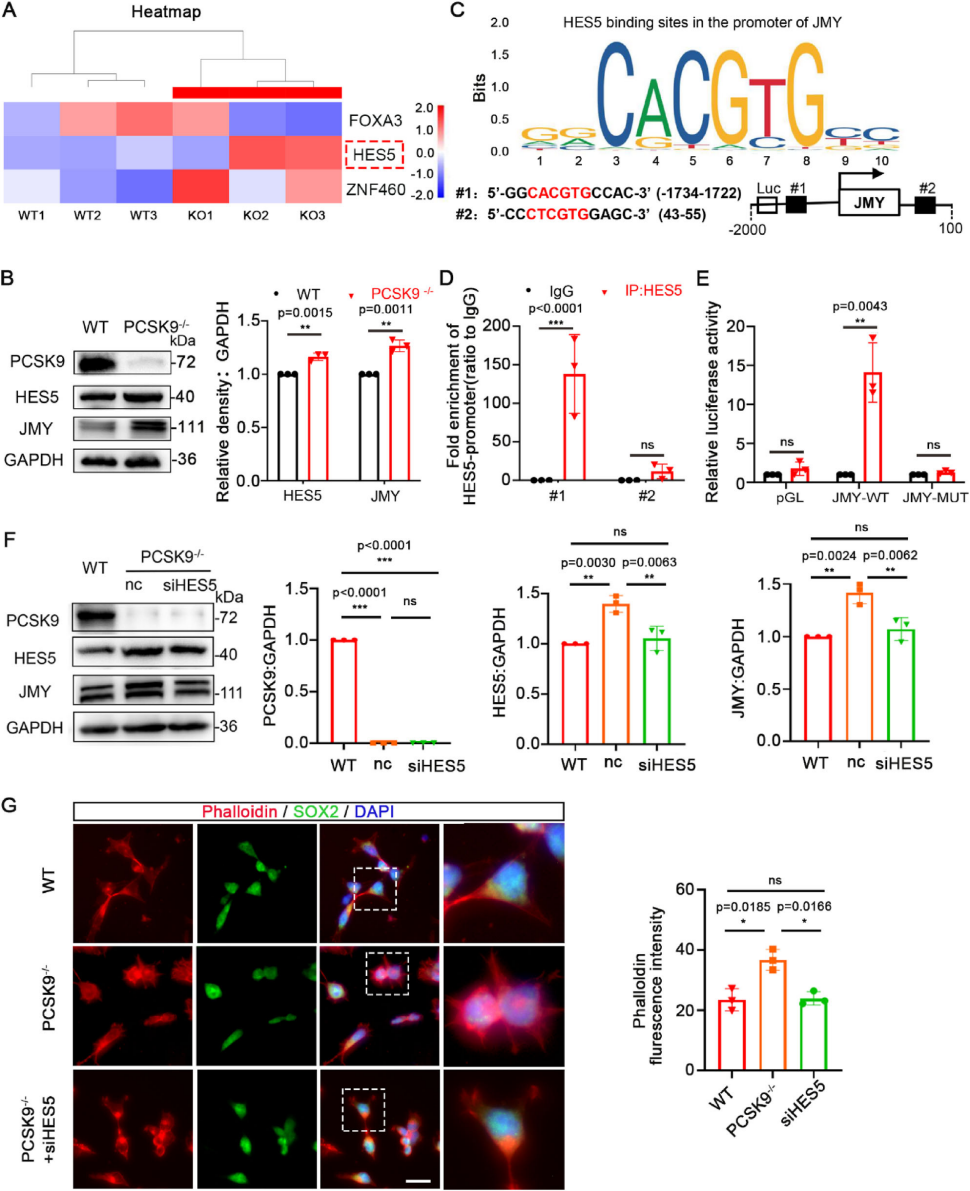
HES5 acts as a transcription factor and regulates JMY expression. A) Heat map of FOXA3, HES5, and ZNF460 expression levels in the transcriptome database. The three rows on the left pertain to the WT groups, and the three on the right to the PCSK9^‐/‐^ groups. Red indicates high expression, and blue indicates low expression. B) WB and quantification of PCSK9, HES5, and JMY protein levels in WT and PCSK9^‐/‐^NOs; n = 3 independent experiments. C) Prediction of HES5–JMY binding sites. D) ChIP‐qPCR analysis of HES5 enrichment at two sites of the JMY promoter; n = 3 individual experiments. E) Luciferase reporter gene experiments verified that HES5 exhibits transcriptional activity against JMY; n = 3 individual experiments. F) WB and quantification of PCSK9, HES5, and JMY protein levels in NPCs after siHES5 rescue experiments; n = 3 individual experiments. G) Immunofluorescence staining and fluorescence intensity quantification of cell microfilaments in WT and PCSK9^‐/‐^ NPCs with and without siHES5 rescue; n = 3 plates from WT, PCSK9^‐/‐^, and PCSK9^‐/‐^ +siHES5; scale bar, 20 µm. Values were mean ± SD. Statistical significance was determined using an unpaired two‐tailed Student's *t*‐test (B, D, E). Statistical significance was determined using one‐way ANOVA with the Dunnet post hoc test (F, G); ns: *P* > 0.05.

We found that JMY is significantly upregulated at the transcriptional level in PCSK9^‐/‐^ NOs. Hence, we performed a preliminary screening using a transcriptomic database to identify the potential transcription factors that regulate JMY. We identified three candidates: FOXA3, HES5, and ZNF460. Notably, HES5 exhibited the highest regulatory potential in the transcriptomic database (**Figure** **4**A). Furthermore, WB analysis showed a concurrent increase in both HES5 and JMY levels in the PCSK9^‐/‐^ group, suggesting that HES5 may positively regulate JMY expression (Figure 4B). Additionally, the JASPAR online database was used to predict the specific binding sites for the transcription factor HES5 on JMY. We identified two potential binding sites: #1 and #2 (Figure 4C). Chromatin immunoprecipitation (ChIP) assays showed significant enrichment of HES5 at JMY promoter sequence #1 (Figure 4D). Further analysis using luciferase reporter assays showed that HES5 overexpression (HES5‐oe) notably enhanced the transcriptional activity of JMY‐WT (Figure 4E).

To substantiate the hypothesis that PCSK9 loss facilitates the upregulation of JMY expression via HES5, we identified the siRNA with the strongest inhibitory effect against HES5. Subsequent rescue experiments corroborated the finding that HES5 positively regulates JMY expression and that HES5 inhibition restores both JMY expression levels and the aberrant morphology of the NPCs (Figure 4F,G). Rescue experiments showed that PCSK9 loss positively modulated JMY expression via HES5.

### 2.5 PCSK9 Impacts NT Structure by Promoting LIN28A Degradation through the Lysosomal Pathway

**Figure 5:**
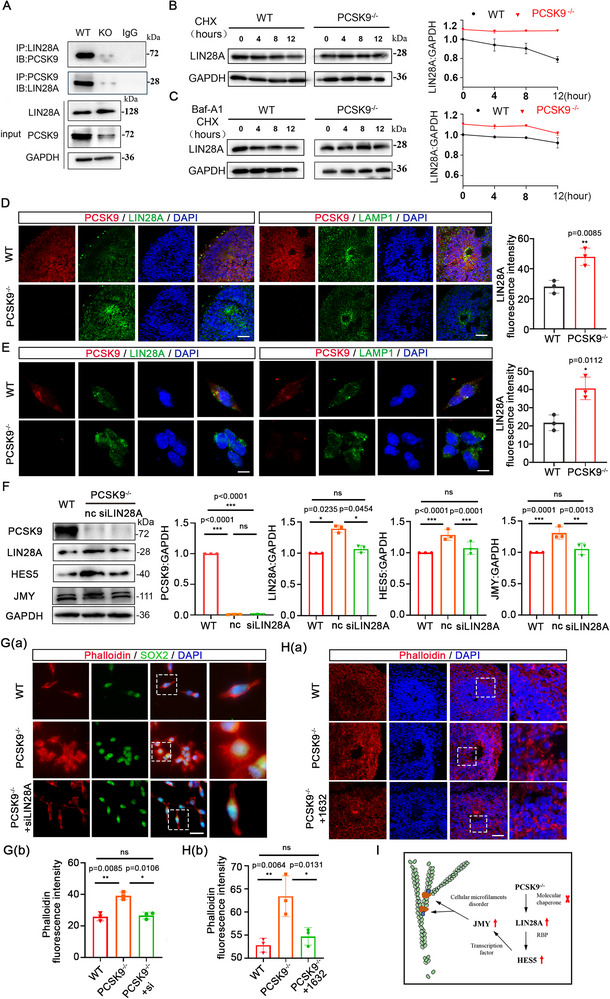
PCSK9 affects NT structure by promoting LIN28A degradation through the lysosomal pathway. A) Co‐IP detection of the interaction between PCSK9 and LIN28A. B) WB and quantification of LIN28A protein levels of NPCs after CHX (10 mM) treatment; n = 3 individual experiments. C) WB and quantification of LIN28A protein levels of NPCs after CHX (10 mm) and Baf‐A1 (20 nm) treatment; n = 3 individual experiments. D,E) Immunofluorescence staining of PCSK9, LIN28A, and LAMP1 (lysosomal marker) in the NT of NOs (D) and NPCs (E); scale bar, 100 µm (D), scale bar, 10 µm (E) F) WB (left) and quantification (right) of PCSK9, LIN28A, HES5, and JMY protein levels in NPCs after siLIN28A rescue; n = 3 individual experiments. G) Immunofluorescence staining a) and fluorescence intensity quantification b) of cell microfilaments in NPCs with and without the siLIN28A rescue; n = 3 plates from WT, PCSK9^‐/‐,^ and PCSK9^‐/‐^ +siLIN28A; scale bar, 20 µm. H) Immunofluorescence staining a) and fluorescence intensity quantification b) of cell microfilaments in NT structure with and without the compound 1632 rescue; n = 3 individual NOs; scale bar, 100 µm. I) Simplified schematic of PCSK9/LIN28A/HES5/JMY regulatory axis‐mediated alteration of cellular microfilament network assembly in NTDs. Values were mean ± SD. Statistical significance was determined using an unpaired two‐tailed Student's *t*‐test (B, C, D, E). Statistical significance was determined using one‐way ANOVA with the Dunnet post hoc test (F, G, H); ns: *P* > 0.05.

Further investigation is warranted to elucidate the mechanism by which PCSK9 regulates HES5. It was reported that PCSK9 may exert its biological functions via lysosomal and proteolytic pathways.^[^ ^24^, ^25^, ^26^ ^]^ Furthermore, PCSK9 functions as a molecular chaperone, facilitating the transport of target proteins to lysosomes for degradation.^[^ ^24^, ^25^, ^26^ ^]^ These findings imply that PCSK9 may play a pivotal role in regulating HES5 through the lysosomal degradation pathway. Protein mass spectrometry analysis conducted following co‐immunoprecipitation (co‐IP) with an anti‐PCSK9 antibody identified that PCSK9 may interact with LIN28A.There, we hypothesize that LIN28A may serve a pivotal role in mediating the interaction between PCSK9 and HES5. Co‐IP experiments showed interaction between PCSK9 and LIN28A with a notable increase in endogenous LIN28A levels following PCSK9 loss (**Figure** **5**A). A comprehensive analysis of peptides from PCSK9 and LIN28A using secondary mass spectrometry showed that certain peptides from LIN28A significantly interacted with PCSK9 (Figure S5A, Supporting Information). Subsequent predictions and analyses using protein structure modeling software showed that PCSK9 and LIN28A could be effectively docked, which suggests the potential for a direct protein structure combination (Figure S5B, Supporting Information). We investigated this further by examining whether PCSK9 induces LIN28A degradation via the lysosomal pathway and found that the lysosomal LIN28A protein levels in the PCSK9^‐/‐^ NPCs were lower than those in the WT NPCs, whereas LIN28A showed high cytoplasmic and total protein levels (Figure S5C,D; Supporting Information). Next, we used cycloheximide (CHX) and bafilomycin A1 (Baf‐A1) to inhibit intracellular protein synthesis and lysosomal degradation, respectively, to investigate the potential physical interactions between PCSK9 and LIN28A within the lysosome. We observed alterations in LIN28A expression in both the WT and PCSK9^‐/‐^ NPCs at various time points after inhibitor treatment. These findings indicate that in the context of inhibited protein synthesis (CHX‐treated), the LIN28A protein levels decreased significantly but at a slower rate in the PCSK9^‐/‐^ NPCs than in the WT NPCs (Figure 5B). In contrast, simultaneous exposure to Baf‐A1, which inhibits lysosomal function, did not significantly reduce LIN28A protein levels between the WT and PCSK9^‐/‐^ NPCs (Figure 5C). These findings suggest that PCSK9 modulates intracellular LIN28A levels via the lysosomal degradation pathway. Furthermore, immunofluorescence staining showed colocalization of PCSK9 with LIN28A and LAMP1 and high total LIN28A level in the PCSK9^‐/‐^ NOs (Figure 5D). We repeated this experiment in the NPCs (Figure 5E) and found that PCSK9 facilitated LIN28A degradation via the lysosomal pathway, with PCSK9 loss resulting in an aberrant increase in LIN28A levels. Subsequent rescue experiments confirmed that PCSK9 loss positively influenced HES5 and JMY expression through LIN28A (Figure 5F). During the PCSK9^‐/‐^ NPC‐induced differentiation, suppression of LIN28A expression substantially ameliorated the morphological abnormalities that were characterized by increased branching in the NPCs (Figure 5G). Subsequently, we validated these findings in the NOs and observed that LIN28A inhibition in PCSK9^‐/‐^ NOs restored the expression of cell microfilaments and most of the NT structures to a ring structure (Figure 5H). Immunofluorescence quantification showed a significant increase in mature neuronal marker levels after LIN28A inhibition compared with those of the PCSK9^‐/‐^ group (Figure S5E, Supporting Information).

Thus, this study shows that the PCSK9 loss influences the organization of the cellular microfilament network via the LIN28A/HES5/JMY regulatory axis, which results in aberrant cell morphology and subsequent malformation of NT structures (Figure 5I).

### 2.6 JMY Aggravates PCSK9 Deficiency‐Induced NTDs in Zebrafish

**Figure 6:**
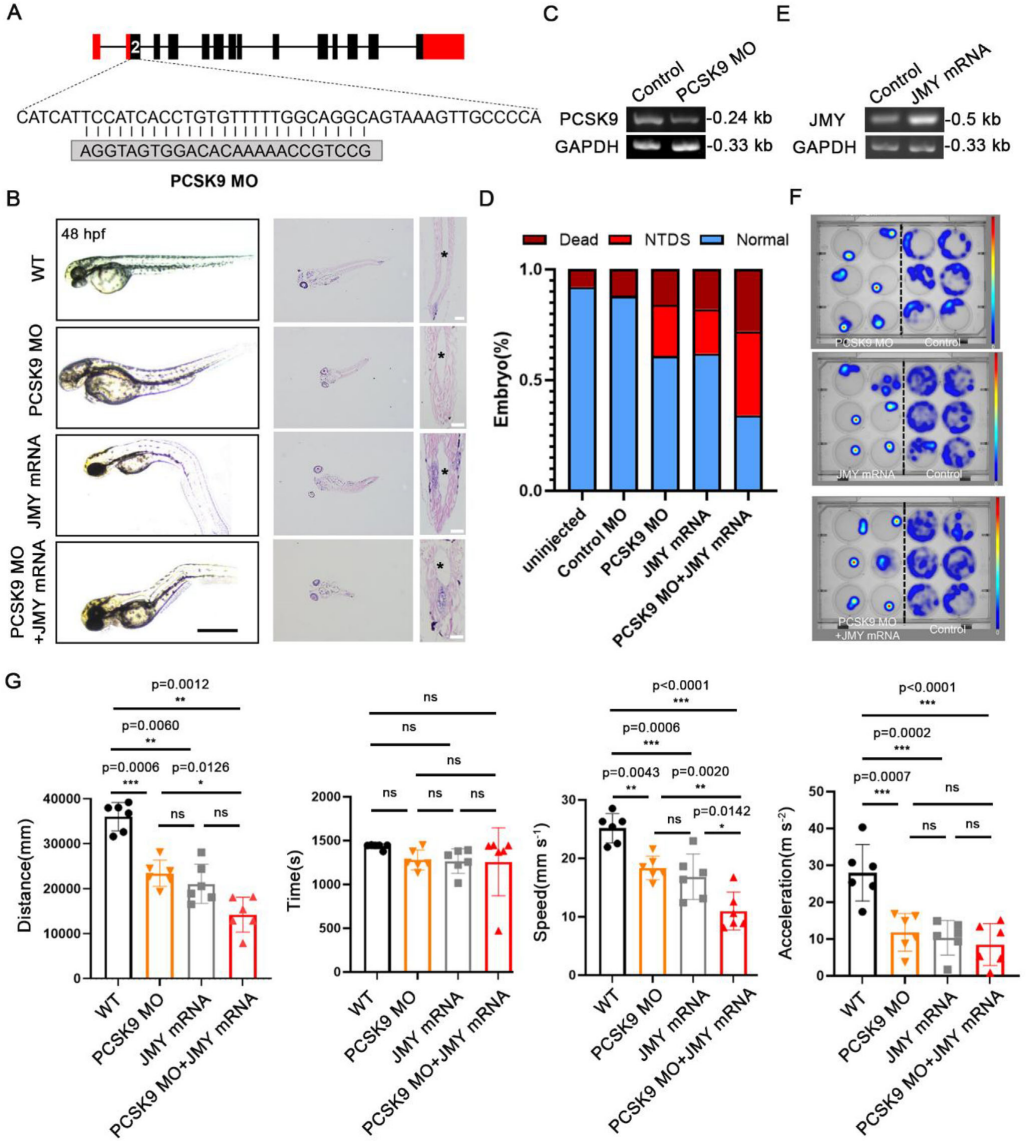
JMY aggravates PCSK9 loss‐induced NTDs in zebrafish. A) Detailed base diagram of the second exon region of zebrafish designed using PCSK9‐MO, resulting in 25 bp depletion. B) Effects of PCSK9‐MO, JMY‐mRNA, and their combined administration on the development of the zebrafish NTs. The left side shows the light microscopy image, and the right side shows a cross‐sectional HE‐stained image and an enlarged NT structure image. “^*^” indicates the neural cavity; scale bar, 1 mm (left) and 50 µm (right). C) RT‐PCR detection of PCSK9 expression after PCSK9‐MO administration. D) Percentage of incidence and mortality of zebrafish NTDs in the PCSK9‐MO, JMY‐mRNA, and combined treatment groups; n = 100 zebrafishes per group. E) RT‐PCR detection of JMY expression after JMY mRNA administration. F) Thermal images of zebrafish activities in the PCSK9‐MO, JMY‐mRNA, and combined administration groups. The right side shows the control group, and the left side shows the deformity group. G) Statistical analysis of zebrafish‐related behaviors in the PCSK9‐MO, JMY‐mRNA, and combined administration groups. The first group shows the distance of movement, the second group shows time, the third group shows speed, and the fourth group shows acceleration; n = 6 individual experiments. Values were mean ± SD. Statistical significance was determined using one‐way ANOVA with the Dunnet post hoc test (G); ns: *P* > 0.05.

To further validate these findings, we used zebrafish as a developmental model to corroborate our conclusions. PCSK9 is ubiquitously expressed during the early development of the ectoderm in both zebrafish and mammals and continues to be expressed throughout neurogenesis.^[^ ^30^ ^]^ Therefore, we aligned the human and rodent PCSK9 cDNA with the zebrafish genome and designed an antisense morpholino (MO) oligonucleotide to target the second exon of PCSK9 to cause a 25‐bp deletion to inhibit endogenous PCSK9 expression (**Figure** **6**A). The PCSK9‐MO homozygous embryos were phenotypically distinguishable from their WT siblings up to 48 h post‐fertilization (hpf), and they exhibited an abnormal NT structure (Figure 6B). We validated the knockdown effect of MO using RT‐PCR to assess the decrease in PCSK9 transcript levels in the PCSK9‐MO‐treated zebrafish (Figure 6C). Although PCSK9‐MO administration was associated with NTDs in zebrafish, the incidence of such deformities was relatively low, with a rate of only 23% (46/200), as shown in Figure 6D.

Furthermore, we investigated whether JMY overexpression in conjunction with PCSK9‐MO administration exacerbated the occurrence of NTDs. We administered JMY mRNA alone, and the zebrafish exhibited NTDs (Figure 6B). RT‐PCR confirmed JMY overexpression in the group that showed deformities (Figure 6E). Combining PCSK9‐MO with JMY mRNA increased the incidence of NTDs to 38% (76/200) with a corresponding increase in mortality (Figure 6D). Furthermore, we assessed the morphology of the NT structure in the embryos by staining tissue sections, which showed shortened NT structure, enlarged lumen, and disorganized neurofilament structure in PCSK9‐MO zebrafish compared with those in WT zebrafish. PCSK9‐MO and JMY mRNA co‐administration exacerbated this effect (Figure 6B). Behavioral studies in zebrafish showed that NTD induction by PCSK9‐MO, JMY‐mRNA, or their combination resulted in significantly restricted movement (Figure 6F). Statistical analysis showed that the onset of NTDs was followed by reduced distance of movement, speed, and acceleration in the affected zebrafish than in the WT zebrafish (Figure 6G). Notably, these parameters were notably low in the group receiving the combination treatment, which suggests that JMY mRNA enhances the incidence and severity of PCSK9‐MO‐induced NTDs.

## 3 Discussion

PCSK9 is a member of the PCSK family and is particularly important for brain development.^[^ ^32^, ^33^, ^34^ ^]^ Analysis of gene databases has shown a significant correlation between functional deletions of PCSK9 and the occurrence of NTDs in humans.^[^ ^28^ ^]^ We previously identified PCSK9 as a biomarker for the prenatal diagnosis of NTDs.^[^ ^29^ ^]^ Furthermore, the lipid‐lowering efficacy of PCSK9 inhibitors, which are widely used in hypercholesterolemia management,^[^ ^35^, ^36^ ^]^ is higher than that of statins. Although current clinical data report no significant association between maternal exposure to these inhibitors and NTD incidence, eight cases of spontaneous abortion have been documented.^[^ ^37^ ^]^ Notably, Vugnier et al. have described a case of high‐dose early pregnancy exposure to alequizumab (a PCSK9 inhibitor), which resulted in isolated fetal agenesis of the corpus callosum detected on routine ultrasound.^[^ ^38^ ^]^ These findings highlight the need for cautious interpretation of safety data. Consequently, the long‐term investigation of the safety of these compounds remains critical, particularly for pregnant women. This study aimed to ascertain whether anti‐PCSK9 drugs interfere with fetal neurodevelopment. Our findings have shown that PCSK9^‐/‐^ESCs fail to form fully developed NT structures during differentiation into NOs. Cerebral organoid database analysis further showed that PCSK9 is enriched in NPCs during early neural development, suggesting that its loss significantly affects NPCs. In fact, PCSK9^‐/‐^ ESCs that differentiated into NPCs exhibited an aberrant morphology with increased cellular branching and dysregulated microfilament expression, which indicates that disrupted microfilament network assembly leads to impaired NT development. Based on these experimental and clinical observations, we suggest enhanced pharmacovigilance in women of childbearing potential—particularly with next‐generation small‐molecule PCSK9 inhibitors (e.g., inclisiran)—to avoid the risks of neurodevelopmental anomalies and pregnancy outcomes.

The primary biological functions of PCSK9 include the regulation and maintenance of lipid homeostasis, neuronal differentiation, and apoptosis.^[^ ^22^, ^23^, ^24^ ^]^ Although PCSK9 has not been directly linked to microfilament dynamics, the transcriptional analyses in this study have identified JMY as a key mediator of PCSK9‐dependent microfilament regulation. JMY enables microfilament assembly by nucleating the Arp2/3 complex, which drives filopodia/lamellipodia formation and shapes cellular morphology.^[^ ^39^, ^40^, ^41^ ^]^ According to these results, PCSK9 loss results in the upregulation of cytoplasmic JMY expression, which affects the assembly of the microfilament network—a process that is essential for the development of the NT structures in the NPCs, thereby leading to an abnormal NT structure. Additionally, PCSK9 loss leads to an aberrant increase in JMY levels in the nucleus and cytoplasm. Notably, JMY knockdown in PCSK9^‐/‐^ ESCs during NPC and NO differentiation restored normal morphology in the majority of cells and rescued the aberrant NT structures in the NOs, which indicates functional reversibility. Complementary zebrafish studies showed that JMY overexpression exacerbated the severity of PCSK9 loss‐associated NTDs, which reinforces its pathological role. Mechanistically, transcriptomic profiling helped identify HES5 as a direct positive regulator of JMY. HES5 is a Notch pathway‐regulated bHLH transcriptional repressor that is critical for neural differentiation.^[^ ^42^, ^43^, ^44^, ^45^, ^46^ ^]^ To the best of our knowledge, this is the first study to implicate HES5 in JMY‐driven NTD pathogenesis. We successfully restored JMY expression and ameliorated most NPC morphological abnormalities by inhibiting HES5 during NTC.

The primary biological role of PCSK9 is to function as a molecular chaperone by selectively binding to members of the LDLR family and facilitating their degradation in lysosomes, which consequently regulates plasma cholesterol levels.^[^ ^23^, ^24^, ^25^, ^26^ ^]^ Therefore, we inferred that PCSK9 may modulate HES5 expression via lysosomal degradation. Although co‐IP experiments did not detect a direct PCSK9–HES5 interaction, subsequent mechanistic studies identified LIN28A as a critical mediator. LIN28A is an RNA‐binding protein that is pivotal for stem cell regulation.^[^ ^47^, ^48^, ^49^ ^]^ It modulates embryogenesis by binding to let‐7 miRNAs via its zinc finger domain^[^ ^50^, ^51^, ^52^ ^]^ and recruits the TUT enzyme to polyuridylate and degrade let‐7, which derepresses downstream targets like HES5.^[^ ^53^ ^]^ The current study has shown that PCSK9 interacts with LIN28A to promote lysosomal degradation. During the differentiation of PCSK9^‐/‐^ NPCs, LIN28A inhibition restored HES5 and JMY expression and normalized the NPC morphology in cell‐based models. Critically, this intervention also rescued the aberrant NT structures in NOs, as evidenced by most NOs exhibiting near‐normal NT structures following LIN28A suppression. The partial morphological rescue observed in both NPCs and NOs may either reflect incomplete siRNA and inhibitor efficacy or imply the presence of additional PCSK9‐dependent regulatory pathways that contribute to NTD pathogenesis. These findings collectively implicate PCSK9 in NT development via the disruption of the LIN28A/HES5 axis disruption, and comprehensive mechanistic studies are required to validate this conclusion.

**Figure 7:**
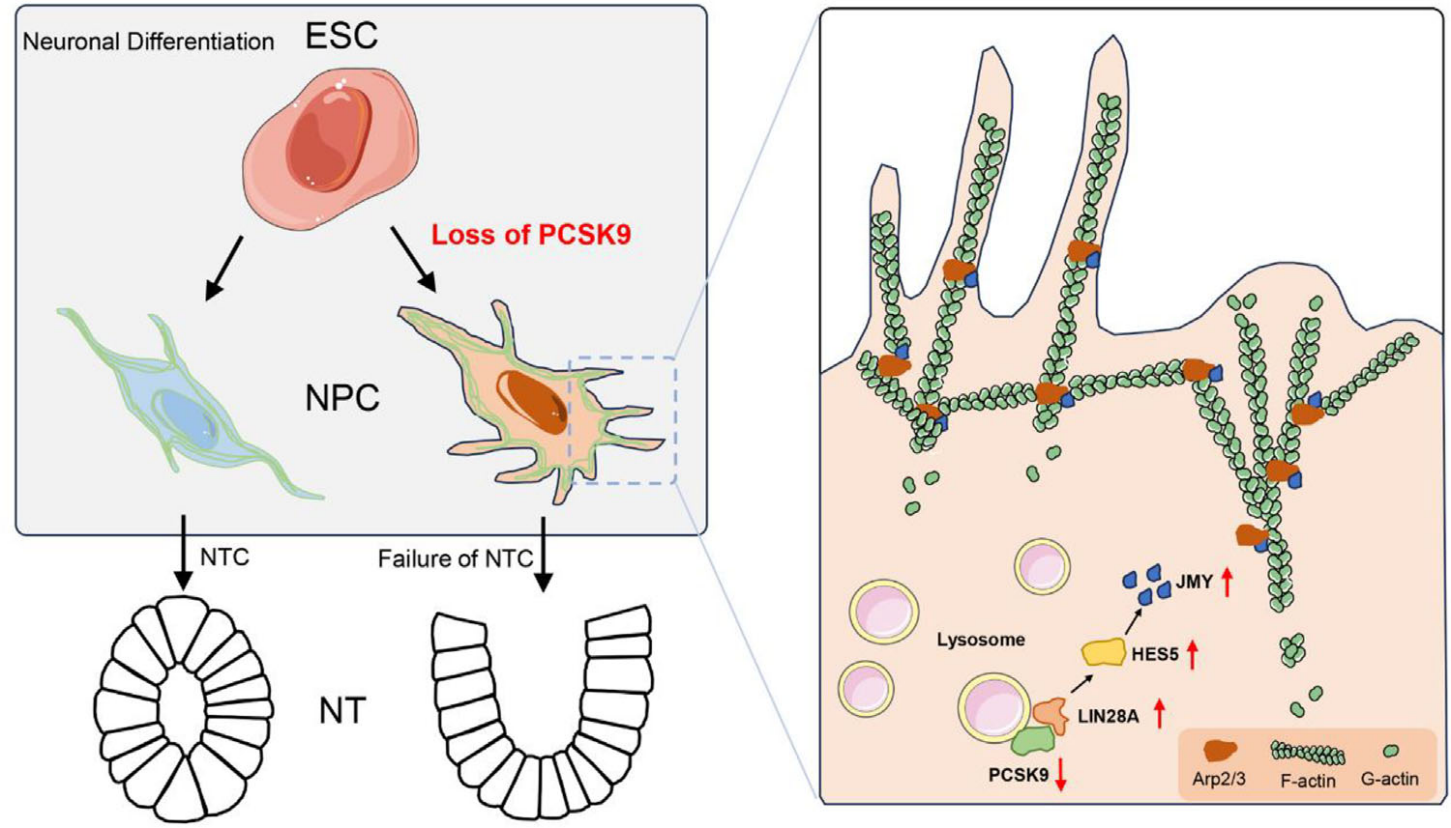
The schematic diagram of our findings. PCSK9 acts as a molecular chaperone that promotes LIN28A degradation via the lysosomal pathway. Furthermore, LIN28A, an RNA‐binding protein, ultimately affects the expression of JMY by regulating the transcription factor HES5. Aberrant elevation of JMY influences the organization of the cellular microfilament network, thereby impacting cell morphology.

In conclusion, this study has elucidated a molecular regulatory network involving PCSK9, LIN28A, HES5, and JMY using a range of biological research methodologies, where PCSK9 was the focal point, and 2D NPCs, 3D NOs, and zebrafish were used as experimental models. This study has elucidated a novel mechanism underlying the influence of PCSK9 on LIN28A expression via the lysosomal degradation pathway, which results in the disruption of cellular microfilament network assembly through the LIN28A/HES5/JMY regulatory axis (Figures 5I and **7**). Ultimately, this process contributes to NTD pathogenesis. The findings of this study offer insights into the potential molecular mechanisms underlying NTDs and establish a theoretical foundation for early diagnosis and treatment of these conditions.

## 4 Experimental Section

### Generation and Characterization of Gene Edited PSCs

ESCs and iPSCs were purchased from the Chinese Academy of Sciences Cell Bank (Shanghai, China). ESCs were fully characterized and cultured on irradiated mouse embryonic fibroblasts (MEFs) in ESC complete culture medium consisting of DMEM/F12, 20% KnockOut Serum Replacement, 1 × GlutaMAX, 1 × MEM non‐essential amino acids (Invitrogen), 0.1 mm 2‐mercaptoethanol(2‐ME), and 10 ng mL^−1^ of human basic FGF (PeproTech). ESCs were seeded onto 6‐well tissue culture plates precoated with MEFs and maintained in ESC complete culture medium. Cells were fed daily and passaged every 4–5 days. iPSCs were cultured in feeder‐free cells on Matrigel‐coated dishes with mTeSR plus medium.

CRISPR‐Cas9‐mediated PCSK9^‐/‐^, PCSK9^R46L,^ and VANGL2^‐/‐^ PSCs were performed by Cyagen (Suzhou, China). Briefly, multiple guide RNAs (gRNA‐A1: AACCTACATTGTGGTGCTGA‐TGG, gRNA‐A2: CCGCCGGGGCTATGTCATCA‐AGG, gRNA‐B1: CTCCATCAGCACCACAATGT‐AGG, gRNA‐B2: CAGACCCGGGGCTGCCCGCCG‐GGG) were theoretically selected for PCSK9^‐/‐^, (gRNA‐A1: CCATGTCGACTACATCGAGG‐AGG, gRNA‐B1: GTGGAGGGGTAATCCGCTCC‐AGG, gRNA‐C1: CTCGGGCACATTCTCGAAGT‐CGG) for PCSK9 ^R46L^ and (gRNA‐A1: GACCAGTGTCCAGCTCCCCG‐AGG, gRNA‐A2: CAGGTTCATGTACACGGTCT‐TGG) for VANGL2^‐/‐^ as the gRNA sequences with the lowest off‐target efficiency, and then mixed with cas9 protein to form ribonucleoprotein (RNP). The RNP complex was transferred to PSCs via electrotransfer. The pool of transfected cells was analyzed by Sanger sequencing to confirm successful editing. The edited pool was used to inoculate single cells for clonal expansion. Each inoculated well was imaged and followed up every 2–3 days to ensure that the population was clonal and descended from a single cell. Two clones were selected and characterized by genotyping using Sanger sequencing, karyotyping using KaryoStat, viability testing, mycoplasma testing, and pluripotency testing.

### Generation of JMY Knockdown Cells

At ≈50% confluence, the cells were incubated with lentiviruses for 12 h. JMY shRNA targeting sequences were used as follows: CTACGACATGAACGGCTGTTA. Next, puromycin was added to the cell cultures for 2 weeks at increasing concentrations of 0.05 µg mL^−1^. Cells were then dissociated into single cells by Accutase treatment for 10 min at 37 °C, and transferred to a flat‐bottom 96‐well plate containing medium supplemented with 5 µm Y27632. After a week, single clones were picked and expanded for 2 weeks.

### NPC Induction and Maintenance

After collecting ESCs from one T75 culture flask, the cell density was adjusted to 5 × 10^4^ mL^−1^ and ≈8000 cells were inoculated in low adhesion 96‐well plates with EB medium (G‐MEM/10% serum medium, 2 mm glutamine, 1 mm sodium pyruvate, 0.1 mm nonessential amino acids, 0.1 mm 2‐ME (10 mm SB431542 and 100 U mL^−1^ PS). At this time, ESC rapidly form cell aggregates called EB. After 2 days of culture, EB were transferred to low‐adhesion 24‐well plates with neural ectoderm induction medium (DMEM/F12, 1% N2 supplement, 1% glutamine, 1% MEM‐NEAA,1 µg m^−1^ heparin, and 100 U mL^−1^ PS), and the fluid was changed every other day. After 3 days of culture, the cell spheres appeared to have neural garland‐like structures, and the culture continued to expand for 4–5 days. The cell spheres were digested into individual cells with accutase digestion solution, and then they were spread on petri dishes to continue the culture. The culture of NPCs was required to be maintained in neural‐inducing medium containing 20 ng mL^−1^ bFGF and 20 ng mL^−1^ EGF, and the medium was changed every other day.

### Generation of NOs

The first three stages of the culture process of NOs were the same as those of NPCs, and the culture was changed to neuroepithelial induction medium at the third stage of culture, that was, inducing differentiation toward the neural ectoderm, and the neuroepithelial‐like structure was formed at 6–8th days of culture. At this time, the cell spheres were embedded in Matrigel gels containing 3‐mm craters, and the gels were solidified by placing them in the incubator for 30 min and then they were blown to the culture dishes, and were added to the mature neural differentiation medium (50% DMEM/F12 mixture and 50% neural basal medium, which contained 0.5% N2 supplement, 1% B27 supplement, 1% glutamine, 1% MEM‐NEAA, 1:4000 insulin, 0.05mM 2‐ME, and 100 U mL^−1^ PS). After being cultured for 9–10th days, the cells were transferred to a rotating bioreactor to continue the expanded culture, and the liquid was changed every 3–4 days. The culture was then transferred to a rotating bioreactor to maintain expansion. NOs were harvested after being cultivated for 30–40th day.

NOs images were generated using light transmission microscopy equipped with a 4 **×** objective and captured using Fiji software for calculating the maximum NO diameters (expressed in µm). The perimeter and the surface areawere computed using formulas: perimeter = πd (expressed in mm) and surface area = πd^2^ (expressed in µm^2^)

### Drug Treatment

During the induction of differentiation into NOs in PCSK9^‐/‐^ ESCs, the NOs was treated with LIN28A inhibitor (compound 1632). NOs were treated with 100 µm compound 1632 started on day 7 of the protocol and continued through until day 35 with fresh drug supplementation every 4 days during medium change.

### Omics Sequencing and Functional Annotation

The three pairs of WT and PCSK9^‐/‐^ NOs that were successfully induced to differentiate were rinsed with pre‐cooled PBS three times for 5 min each, and then the NOs were sliced into small pieces (≤ 0.5 mm) as much as possible with a sterile blade. Then, the three treated pairs of NOs were stored in labeled 2 mL freezing tubes and immediately placed in liquid nitrogen for 3–4 h. Finally, dry ice was transported to Biomarker Technologies (Beijing, China) for subsequent transcriptomic sequencing. RNA‐seq libraries were prepared using Novogene and sequenced on an IlluminaHiSeq4000 system. Differential expression analysis was performed using DESeq, and heat maps were generated using iDEP.

All data analyses were annotated to DEGs based on the Nr, Pfam, COG, Swiss‐Prot, and KEGG Ortholog databases. Target DEGs were analyzed using KEGGPathway6 and GO enrichment analyses.

### Immunostaining and Immunohistochemical Staining

For NPC immunostaining, cells were fixed in 4% PFA for 15 min, washed three times with PBS, and then incubated in blocking buffer (PBS containing 2% goat serum, 1% BSA, and 0.1% TritonX‐100) with primary antibody overnight at 4 °C, followed by incubation with the secondary antibody for 1 h at room temperature, and then blocked by DAPI staining for 5 min, followed by fixation in 4% PFA for 30 min, dehydration in sucrose overnight at 4 °C, and finally encapsulation in gelatin, followed by freezing on dry ice. Frozen NOs were cut into 10 µm thick sections for immunohistochemical staining. The specific primary antibodies used are listed in Table S1 (Supporting Information). The secondary antibodies used were Alexa 488 and Alexa 555 conjugated to specific IgG types. All experiments were repeated at least three times, and representative images were shown in individual figures. Images were captured using ZeissLSM700 or ZeissLSM980 confocal microscopy. To present the overall staining results of the NOs, large‐field images were acquired using a confocal microscope equipped with ZEN software (Carl Zeiss). Imaging was performed at 10 × magnification using a tiled scanning approach. The individual tile images were automatically stitched into a composite image using the integrated stitching module within the ZEN software. Sample images were prepared using ImageJ and Photoshop software. Immunofluorescence intensity was quantified under consistent imaging parameters (exposure time: phalloidin, 200 ms; Lin28A, 400 ms). Background signals from adjacent non‐immunofluorescent regions were subtracted using ImageJ (v1.53, NIH), and the mean intensity of regions of interest was calculated.

### Reverse Transcription Quantitative Polymerase Chain Reaction (RT‐qPCR)

Total RNA was extracted from NPCs or NOs using TRIzol reagent and reverse transcribed using the HiScript Q RT SuperMix for qPCR kit, according to the manufacturer's instructions. The obtained cDNA was mixed with AceQ qPCR SYBR green master mix and gene‐specific primers in a StepOnePlus instrument (Applied Biosystems, California, USA) for RT‐qPCR. The cycle time values were normalized to glyceraldehyde‐3‐phosphate dehydrogenase for the same samples. Relative amounts were calculated using the 2^‐ΔΔCT^ method. Primer sequences used for qPCR were listed in Table S1 (Supporting Information).

### Cell Transfection

At each stage of ESC‐induced differentiation into NPCs, siRNAs targeting JMY, HES5, and LIN28A (GenePharma, Shanghai, China) (Table S2, Supporting Information) were transfected into cells using Lipofectamine RNAiMAX in the medium according to the manufacturer's instructions for 8 h. After the mixture was removed, the cells were cultured in the medium.

### Western Blotting

NPCs or NOs were lysed in a radioimmunoprecipitation assay buffer containing a protease inhibitor mixture. The lysates were centrifuged at 16000 × g for 15 min at 4 °C, and the protein concentration in the supernatant was determined using the bicinchoninic acid method according to the manufacturer's instructions. Cytosolic and lysosomal protein extractions were performed according to the Beyotime (Shanghai, China) and Solarbio (Beijing, China) kit instructions. After quantification of the extracted proteins, proteins (30 µg) were separated by 10% SDS polyacrylamide gel electrophoresis, transferred to polyvinylidene difluoride membranes, and immunoblotted with antibodies against PCSK9 (1:1000), JMY (1:1000), HES5 (1:1000), LIN28A (1:1000), LAMP2 (1:500), and GAPDH (1:5000). After washing, the membranes were coupled with the corresponding horseradish peroxidase‐coupled secondary antibodies for 1 h, and the blots were analyzed using an ImageQuant LAS 4000 imaging system (GE Healthcare, USA) and a Bio‐Rad Gel Doc XR documentation system.

### Single‐Cell Transcriptome Bioinformatics Analysis

The public database Single Cell PORTAL platform (www.singlecell.broadinstitute.org) was searched for cerebral organoids and found the classes of “human cerebral cortex” constructed at different developmental time points on the platform.^[^ ^54^ ^]^ PCSK9 was searched on the organ data platform, observed the degree of aggregation and cell type of PCSK9 enrichment in the cerebral organoids at different developmental time points, and statistically analyzed the relevant data.

### Coimmunoprecipitation (co‐IP)

NPCs were lysed in IP lysis buffer on ice for 30 min, and the lysates were centrifuged at 12 000 rpm at 4 °C for 10 min. Primary antibodies (anti‐PCSK9 or anti‐LIN28A) and protein A/G agarose beads were added to the supernatant, and the samples were incubated at 4 °C overnight. The next day, the beads were washed six times with the IP lysis buffer. After resuspension in 1 × SDS‐PAGE buffer, beads were boiled for 10 min. The next step for WB validation or mass spectrometry identification of the proteins was performed and analyzed by Bioprofile Co. Ltd (Shanghai, China).

### Chromatin Immunoprecipitation Assay (ChIP)

The specific procedure for ChIP was performed according to the manufacturer's instructions. Briefly, NPCs were fixed with 1% formaldehyde solution and lysed with a cell lysis solution. The isolated nuclear extracts were sheared through 20 sonication cycles (30 s on and 30 s off per cycle). DNA gel electrophoresis was then performed to verify that the isolated chromatin was sheared into small fragments of ≈300–800 bp. The magnetic beads were pre‐purified with a wash solution and rotationally incubated with the HES5 antibody at 4 °C for 1 h. Isolated chromatin was then incubated with the prepared magnetic beads at 4 °C overnight. Immunoprecipitated chromatin was repeatedly washed and then added with 0.2 mol L^−1^ NaCl at 65 °C overnight. The purified DNA was used on the third day and recovered. Finally, DNA was quantified using qPCR.

### Dual Luciferase Reporter Assay

Two sequences of 2000 and 1000 bp upstream of JMY exon 1 were inserted into plasmid pGL4.18 and transfected them into NPC to screen for stable cell lines. Then, pEGFP‐JMY‐WT, pEGFP‐JMY‐MUT, or empty vector were co‐transfected into NPC for 48 h. Luciferase activity was detected using the Dual Luciferase Reporter Gene Assay System with the procedure described by the manufacturer (Promega, Madison, WI). Change of luciferase activity in treated cells was expressed as a percentage of the controls.

### Protein 3D Structure Prediction and Interaction Analysis

The protein structure data for PCSK9 and LIN28A were retrieved from the protein data platform RCSB PDB (www.rcsb.org). PCSK9 (2pmw) and LIN28A (5udz), Discovery Studio 2019 Client, and PyMol software were used to perform protein structure simulation and protein molecular interaction analysis, as well as the ionic bonds and hydrogen bonds of internal molecules.

### Zebrafish Maintenance and Microinjection of Morpholino Antisense Oligonucleotides

The animal study protocol was approved by the Administrative Committee on Animal Research in Shengjing Hospital of China Medical University (the protocol code was 2020PS153K(X1)). The TU strain of zebrafish was maintained and propagated according to standard procedures. A zebrafish culture system (ESEN Environ Science, Beijing, China) was used to control the temperature and diurnal cycle. All embryos were fertilized and incubated at 28.5 °C.

The antisense oligonucleotide morpholino (MO) against PCSK9 designed in this study was designed and obtained by Gene Tools, LLC. The sequence targeting the 5′ untranslated region and translation start region of the second exon of the PCSK9 mRNA was 5′‐GCCTGCCAAAAACACAGGTGATGGA‐3′. The standard control for the experiment was the sequence 5′‐GTATTGAGGTCGTCATCCATCATCC‐3′. All oligonucleotides were diluted with distilled water and stored at −20 °C.

For JMY mRNA synthesis, capped positive‐sense RNA was synthesized using the mMessage mMachine in vitro transcription kit (Ambion, Austin, TX, USA), according to the manufacturer's instructions.

The synthesized RNA was diluted to an appropriate concentration using RNase‐free water. In all experiments, MO and mRNA were suspended in water containing phenol red (0.05%). Approximately 1–3 nL of the solution (10 mm) was injected into fertilized wild‐type zebrafish eggs (at the one‐ to two‐cell stage) using the InjectMan4 microinjection system (Eppendorf, Germany).

### Zebrafish Behavioral Analysis and Histochemical Staining

The Animal Trajectory Tracking System (Noldus, The Netherlands) was used to analyze zebrafish‐related behavioral science (including action thermograms, swimming time, and swimming distance). Prior to the experiment, zebrafish were placed in a six‐well plate, and after they were acclimatized to the plate, they were placed in the observation system. The camera in the observation system directly recorded the spontaneous activity of zebrafish in the hole plate for 30 min. EthoVision XT version 17 software was used to calculate zebrafish activity trajectories and distances and to quantify the average swimming speed (mm s^−1^) and acceleration (m s^−2^) of the zebrafish. Investigators were blinded to all treatment groups in behavioral tests.

After testing, zebrafish were fixed in 4% PFA at 4 °C for 24 h. The samples were then embedded in resin according to standard procedures. Cross sections (0.5 µm) were obtained using a Leica RM2255 slicer (Wetzlar, Germany). HE staining was performed according to standard procedures. Imaging was performed using a Nikon Eclipse Ni compound microscope (Tokyo, Japan) equipped with a camera.

### Statistical Analysis

All data were analyzed using the GraphPad Prism software (version 9.0). Each experiment was performed in triplicate. The unpaired Student's *t*‐test was used to compare the differences between the two groups. One‐way and two‐way analysis of variance (ANOVA) were used to compare multiple groups. ns, not significant; ^*^, *p* < 0.05; ^**^, *p* < 0.01; ^***^, *p* < 0.001.

### Antibodies and Reagents

All reagents, commercial kits, and antibodies are listed in Table S3 (Supporting Information).

## Conflict of Interest

The authors declare no conflict of interest.

## Supporting information



Supporting Information

## Data Availability

The data that support the findings of this study are available from the corresponding author upon reasonable request.
