# Supplementary material for: PCSK9 Loss‐of‐Function Disrupts Cellular Microfilament Network via LIN28A/HES5/JMY Axis in Neural Tube Defects
Source: Adv Sci (Weinh). 2025 Aug 11;12(41):e04291. doi: 10.1002/advs.202504291 (PMC12591174; doi:10.1002/advs.202504291)
Supplement: Supplementary file 1 — Supporting Information [file ADVS-12-e04291-s001.pdf]

PCSK9 Loss-of-Function Disrupts Cellular Microfilament Network via LIN28A/HES5/JMY Axis in Neural Tube Defects

*Xiaoshuai Li, Rui Wang, Wenting Luo, Hui Gu, Tianchu Huang, Qiushi Wang, Zhengwei Yuan**

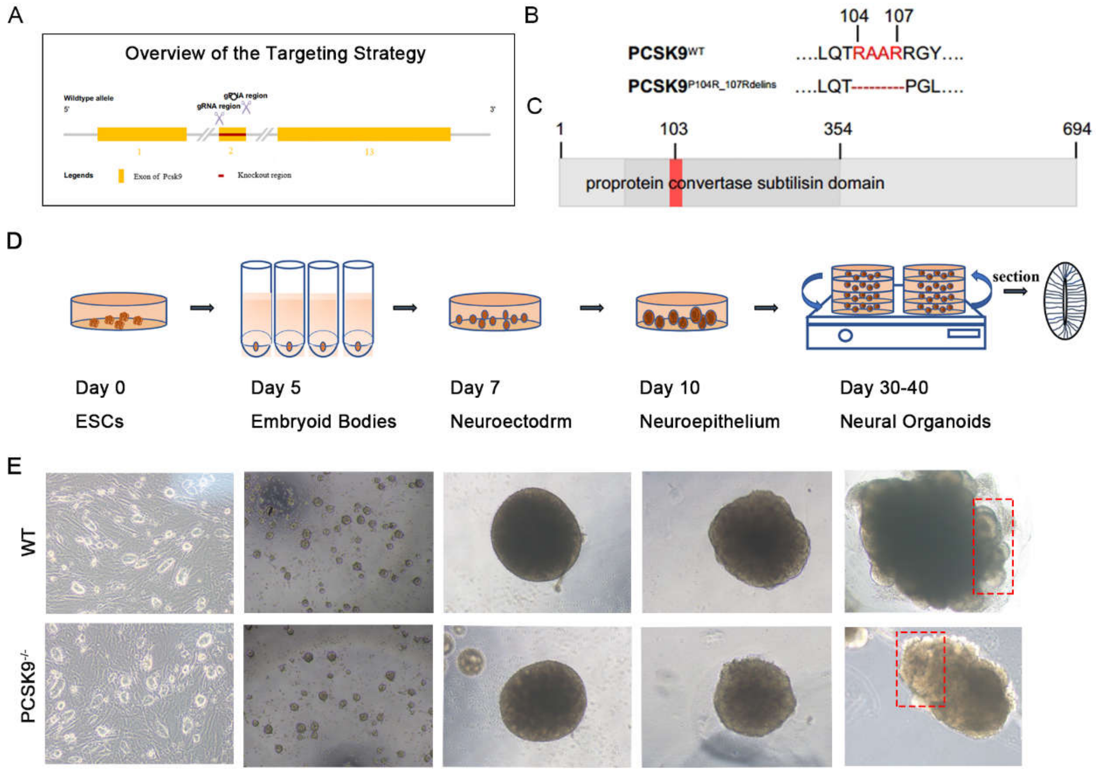

Figure S1. Construction of PCSK9$^{-/-}$ NOs. A) Overview of the PCSK9 knockout strategy. B) PCSK9 amino acid knockout site map. C) PCSK9 protein knockout site map of domain changes. D) NOs-induced culture model map. E) Representative images of WT and PCSK9$^{-/-}$NOs-induced culture light microscopy; scale bar, 200μm.

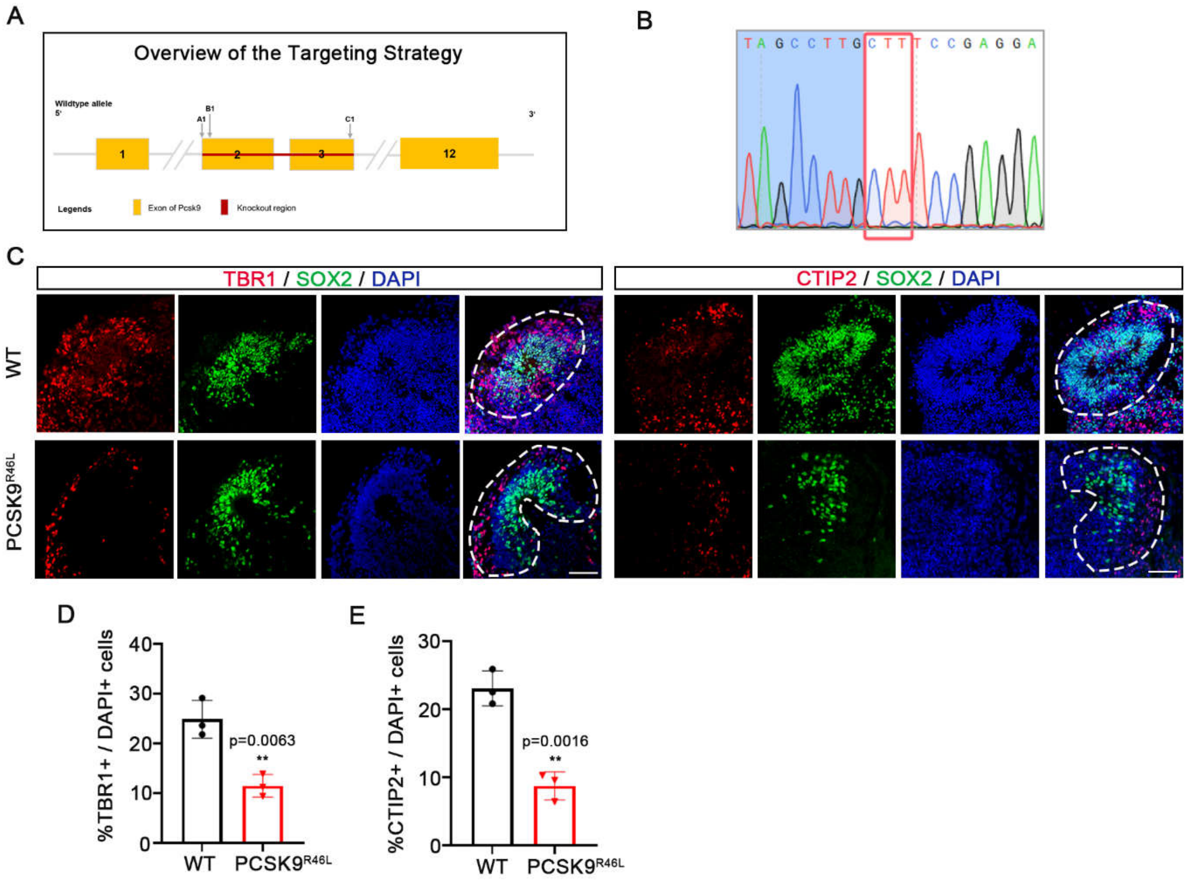

Figure S2. PCSK9^{R46L} leads to a decrease in the expression of mature neurons in NT structure of NOs. A) Overview of the targeting strategy of PCSK9^{R46L} mutation. B) CRISPR/Cas9-mediated gene editing of PCSK9^{R46L} point mutation in iPSCs, resulting p.R46L(CGT to CTT) mutation. C-E) Immunofluorescent staining and quantification of TBR1+/CTIP2+ mature neurons in WT and PCSK9^{R46L} NT structure of NOs. The white circular or irregular shape represents the VZ region indicating the borders of VZ structures; n = 3 individual NOs; scale bar, 100µm. Values were mean ± SD. Statistical significance was determined using unpaired two-tailed Student's t-test (D, E).

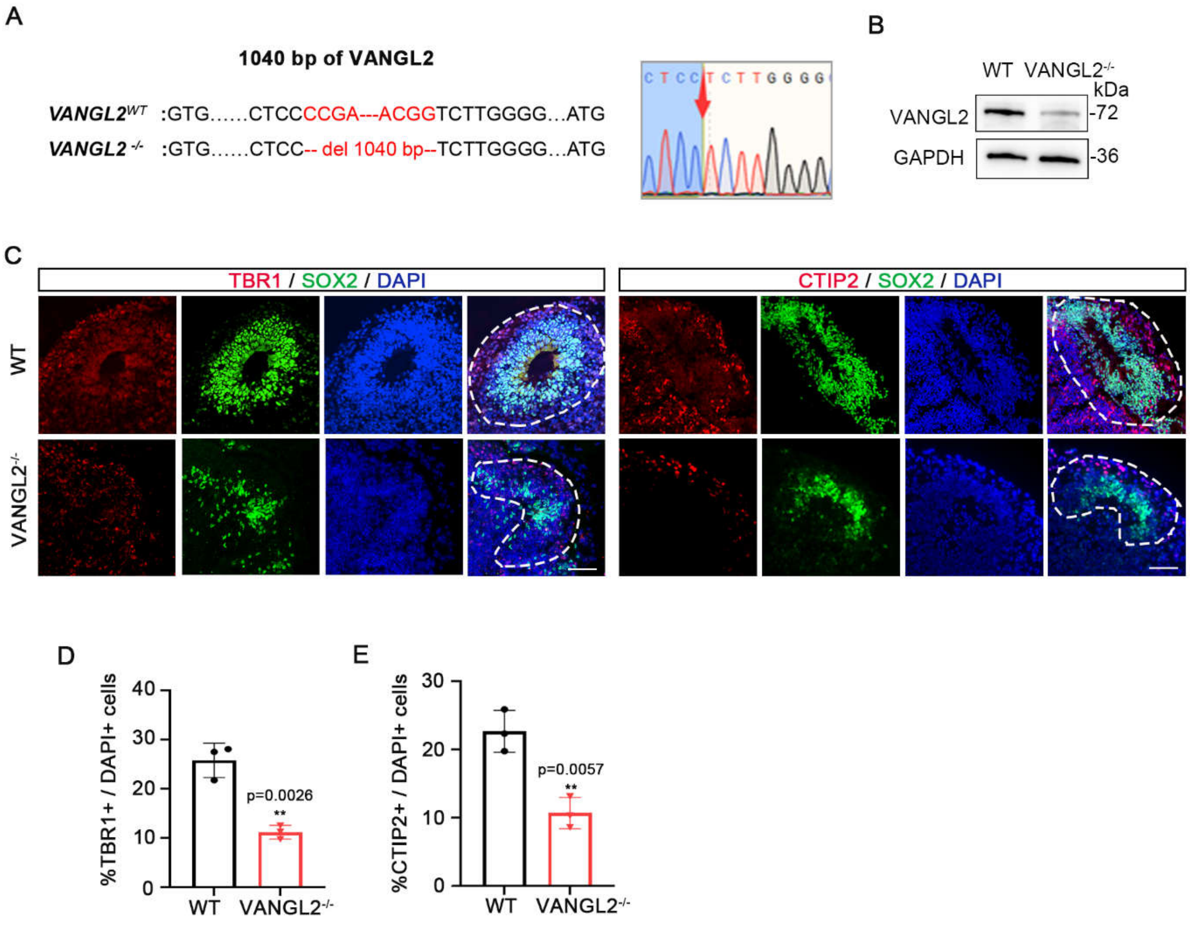

Figure S3. Loss of VANGL2 leads to a decrease in the expression of mature neurons in NT structure of NOs. A) CRISPR/Cas9-mediated gene editing of VANGL2 locus in iPSCs, resulting in 1040 bp depletion. B) WB of VANGL2 expression in WT and VANGL2$^{-/-}$ iPSCs. C-E) Immunofluorescent staining and quantification of TBR1+/CTIP2+ mature neurons in WT and VANGL2$^{-/-}$ NT structure of NOs. The white circular or irregular shape represents the VZ region indicating the borders of VZ structures; n = 3 individual NOs; scale bar, 100μm. Values were mean ± SD. Statistical significance was determined using unpaired two-tailed Student's t-test (D, E).

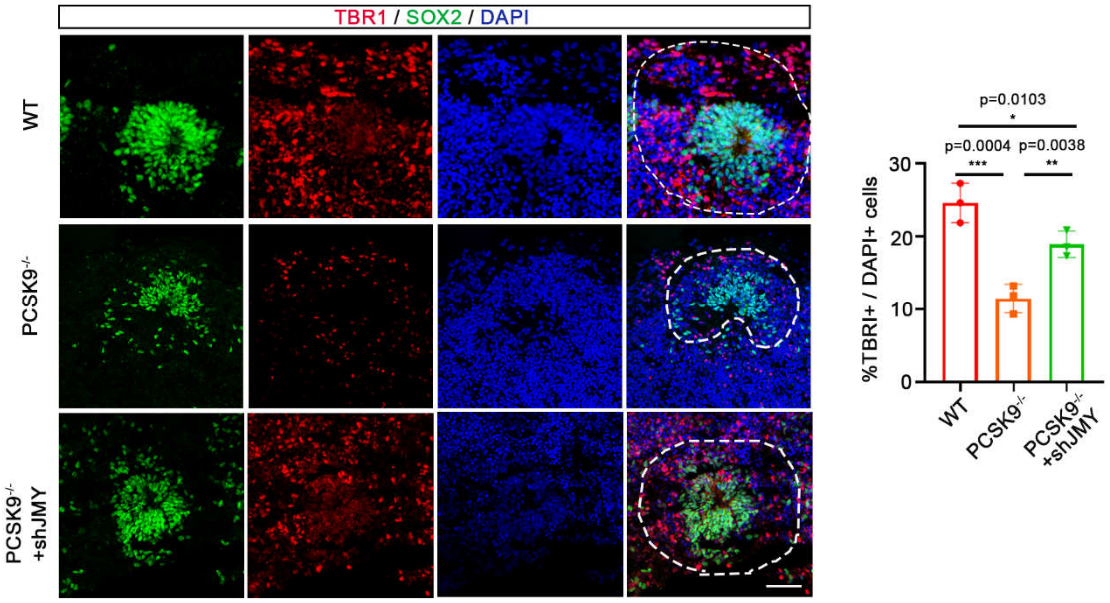

Figure S4. Knockdown of JMY expression restores mature neurons in PCSK9$^{-/-}$ NT structure of NOs. Immunofluorescent staining and quantification of TBR1+ mature neurons in WT and PCSK9$^{-/-}$ NT structure of NOs with and without shJMY rescue. The white circular or irregular shape represents the VZ region indicating the borders of VZ structures; n = 3 individual NOs; scale bar, 100µm. Statistical significance was determined using one-way ANOVA with the Dunnet post hoc test.

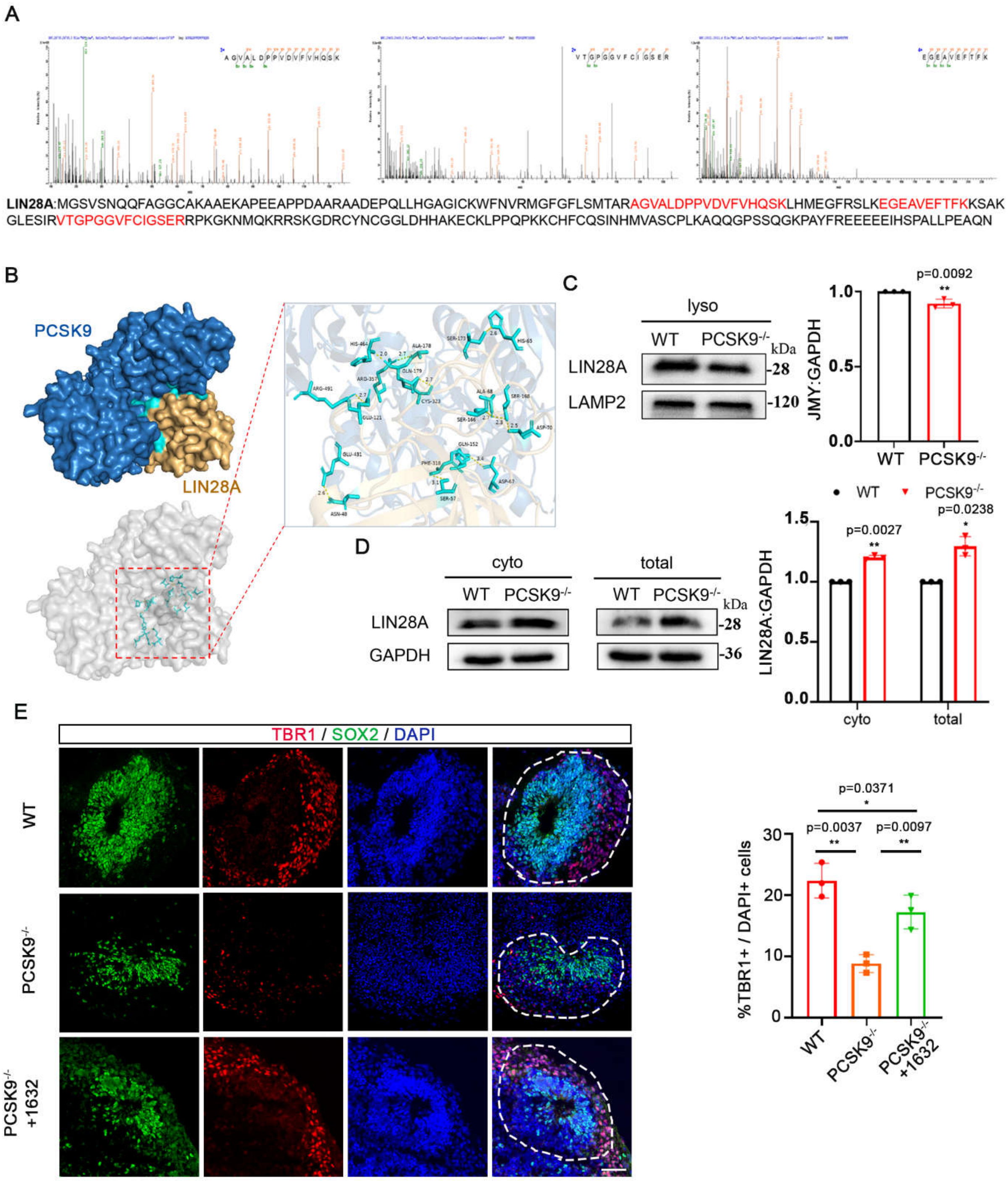

Figure S5. PCSK9 affects NT structure by promoting LIN28A degradation through the lysosomal pathway. A) Secondary mass spectrometry results of the structure peptides of PCSK9 and LIN28A. The three mass spectrometry results corresponded to the red amino acid sequences shown below. B) Molecular protein software simulates the docking results of the PCSK9 and LIN28A proteins. Dark blue indicates the three-dimensional structure of the PCSK9 protein, yellow indicates the three-dimensional structure of the LIN28A protein, light blue indicates the amino acid at the docking position, and the red dotted box indicates the amino acid and hydrogen bond for specific interactions. C, D) WB and quantification of LIN28A protein levels

in lysosomes (up) and cytoplasm (down); n = 3 individual experiments. E) Immunofluorescent staining and quantification of TBR1+ mature neurons in WT and PCSK9$^{-/-}$ NT structure of NOs with and without the compound 1632 rescue. The white circular or irregular shape represents the VZ region indicate the borders of VZ structures; n = 3 individual NOs; scale bar, 100μm. Values were mean ± SD. Statistical significance was determined using unpaired two-tailed Student's t-test (C, D). Statistical significance was determined using one-way ANOVA with the Dunnet post hoc test (E).

Table S1

Primers used for RT-qPCR

	Forward	Reverse
PCSK9	TACCACACGTTTCAATCCACCAAT	CCCCTCTGTCCTCCTATGATTGTA
JMY	GAAGGGGTTAAACACCTCTGC	ATGCTCTGCTTAAACACAATCCT
PAK5	CCGTGACTCGCTCCAACTCTC	TTGTGTAGTCCGCAGTCGTATCG
DNAH5	GAAGCCGCACTCCAGACCATC	GAAACAGCAGCAGCACACAGTC
WDR62	AAAGGGTGCTGGACAAGTGGATC	GTTGGTGAGGTAGAGTAGGCTGTG
GAPDH	ACCACAGTCCATGCCATCAC	TCCACCACCCTGTTGCTGTA

Table S2

siRNA duplexes used to knockdown specific proteins

Gene		Base sequence
Control	sense	UUCUCCGAAGGUGUCACGUTT
	antisense	ACGUGACACCUUCGGAGAATT
JMY	sense	GGGCAAGUCAUCCUUAAGUTT
	antisense	ACUUAAGGAUGACUUGCCCTT
HES5	sense	GUCAGCUACCUGAAACACATT
	antisense	UGUGUUUCAGGUAGCUGACTT
LIN28A	sense	GGUGUGUUCUGUAUUGGGATT
	antisense	UCCCAAUACAGAACACACCTT

Table S3

Reagents, commercial kits, and antibodies

Reagent or Resource	Source	Cat
Dulbecco's modified eagle medium (DMEM)	Gibco	12100-046
DMEM/F12 medium	Gibco	12500-062
mES complete culture medium	Cell Bank of Chinese Academy of Sciences	SCSP-603
neurobasal medium	Gibco	21103-049
mTeSR1 plus	StemCell	100-0276
Fetal bovine serum (FBS)	Gibco	16000-044
Penicillin-Streptomycin	NCM Biotech	C125C5
sodium pyruvate	Gibco	11360-070
MEM-non-essential amino acids (MEM-NEAA)	Stemcell	7600

N2 supplement	Stemcell	7152
B27 supplement	Gibco	17504044
SB431542	Stemcell	72234
glutamin	Stemcell	7100
Insulin solution	Merck	I9278
2-Mercaptoethanol	Merck	8057400005
Lin28-let-7 antagonist 1 (compound 1632)	MedChemExpress	HY-112659
trypsin	Solarbio	9002/7/7
EDTA	Gibco	1948/12/25
BCA protein assay	KeyGen BioTECH	KGP903
Lipofectamine RNAi MAX	Invitrogen	13778-150
Lipofectamine 3000	Invitrogen	L3000-15
TRIzol reagent	Life Ambion	15596018
HiScript® III RT SuperMix for qPCR	Vazyme Biotech	R323-01
AceQ qPCR SYBR green master mix	Vazyme Biotech	Q341-02/03
Protease Inhibitor	Invitrogen	A32965
PVDF	Millipore	IPVH00010
BeyoECL Moon	Beyotime Biotechnology	P0018FM
DAPI	Beyotime Biotechnology	P0131
Phalloidin	Absin	abs47048273
Membrane and Cytosol Protein Extraction Kit	Beyotime Biotechnology	P0033
Lysosomal protein extraction kit	Solarbio Life Science	EX1230
SimpleChIP® Enzymatic Chromatin IP Kit (Magnetic Beads)	Cell Signaling Technology	9003
ChIP-Grade Protein G Agarose Beads	Cell Signaling Technology	9007
**Antibody**		
PCSK9	proteintech	55206-1-AP
LIN28A	proteintech	11724-1-AP
HES5	proteintech	22666-1-AP
JMY	proteintech	25098-1-AP
SOX2	R&D Systems	MAB2018
TBR1	Abcam	ab31940
CTIP2	Abcam	ab18465
VANGL2	proteintech	21492-1-AP

LAMP1	Abcam	ab24170
LAMP2	Abcam	ab13524
anti-mouse IgG (H+L), HRP conjugated	Proteintech	SA00001-1
anti-rabbit IgG (H+L), HRP conjugated	Proteintech	SA00001-2
anti-rabbit Alexa Fluor 488	Invitrogen	A11008
anti-rabbit Alexa Fluor 488	Invitrogen	A21422
